# Fully automated web-based tool for identifying regulatory hotspots

**DOI:** 10.1186/s12864-020-07012-z

**Published:** 2020-11-18

**Authors:** Ju Hun Choi, Taegun Kim, Junghyun Jung, Jong Wha J. Joo

**Affiliations:** 1 Department of Computer Science and Engineering, Dongguk University-Seoul, Seoul, 04620 South Korea; 2 Department of Life Science, Dongguk University-Seoul, Seoul, 04620 South Korea

**Keywords:** Regulatory hotspot, Parallel processing, Web-based tool, Google cloud compute engine (GCE), PLINK, VCF (variant call format), eQTL

## Abstract

**Background:**

Regulatory hotspots are genetic variations that may regulate the expression levels of many genes. It has been of great interest to find those hotspots utilizing expression quantitative trait locus (eQTL) analysis. However, it has been reported that many of the findings are spurious hotspots induced by various unknown confounding factors. Recently, methods utilizing complicated statistical models have been developed that successfully identify genuine hotspots. Next-generation Intersample Correlation Emended (NICE) is one of the methods that show high sensitivity and low false-discovery rate in finding regulatory hotspots. Even though the methods successfully find genuine hotspots, they have not been widely used due to their non-user-friendly interfaces and complex running processes. Furthermore, most of the methods are impractical due to their prohibitively high computational complexity.

**Results:**

To overcome the limitations of existing methods, we developed a fully automated web-based tool, referred to as NICER (NICE Renew), which is based on NICE program. First, we dramatically reduced running and installing burden of NICE. Second, we significantly reduced running time by incorporating multi-processing. Third, besides our web-based NICER, users can use NICER on Google Compute Engine and can readily install and run the NICER web service on their local computers. Finally, we provide different input formats and visualizations tools to show results. Utilizing a yeast dataset, we show that NICER can be successfully used in an eQTL analysis to identify many genuine regulatory hotspots, for which more than half of the hotspots were previously reported elsewhere.

**Conclusions:**

Even though many hotspot analysis tools have been proposed, they have not been widely used for many practical reasons. NICER is a fully-automated web-based solution for eQTL mapping and regulatory hotspots analysis. NICER provides a user-friendly interface and has made hotspot analysis more viable by reducing the running time significantly. We believe that NICER will become the method of choice for increasing power of eQTL hotspot analysis.

## Background

Regulatory hotspots are genetic variations that regulate the level of expression of thousands of genes [1–4]. Finding hotspots can guide us to understand the causes and mechanism of many complex diseases and traits [5]. Thus far, genetic studies have been focused on finding regulatory hotspots using eQTL mapping. However, recent studies have reported that many of the previously identified hotspots do not replicate and that these are “spurious hotspots” induced by various unknown confounding effects such as non-biological effects during sample preparation and expression measurements. Several methods have been proposed to remove these confounding effects [6–9].

Next-generation Intersample Correlation Emended (NICE) [10] is one such method that has been reported to show superior sensitivity and specificity compared to others. In spite of its advantages, the NICE program is not widely used because of two main drawbacks. NICE is neither very user friendly nor very efficient in terms of the computing performance. It is not user friendly because prior to executing a NICE program, users are required to install several specific packages, create a script file, and perform self-debug. It is also impractical to run NICE on real datasets, as most of the eQTL datasets contain hundreds of thousands of SNPs (Single Nucleotide Polymorphisms), and hotspot analysis tools such as NICE use very complicated statistical models.

To resolve these issues, we introduce a fully automated hotspot analysis program referred to as NICE-Renew (NICER). NICER is created as a web-based tool, where users can activate an analysis by simply clicking and uploading data on the web browser. Besides, users may run NICER on Google Cloud Compute Engine or even download and install the web software as docker image to run NICER web service on their local computers or servers, in which their private data can be processed using their own resources. NICER has enhanced computing performance by incorporating multi-processing concepts in the program, for which the running time reduces linear to the number of processes used. In addition, NICER allows different data formats and provides some visualization tools, which help users better understand their result and provide a fast, more meaningful view in context.

Utilizing a yeast dataset, we show that NICER can be successfully used in an eQTL analysis to identify 29 genuine regulatory hotspots, of which 17 were previously reported elsewhere.

### Implementation

In this paper, we introduce a fully automated hotspot analysis tool referred to as NICER, which provides enhanced NICE in the form of a website as well as in the form of downloadable software. The website and the documentation of the program can be accessed from the NICER website:

URL http://cblab.dongguk.edu/NICER/NICE_index.jsp

### Fully-automated web-based program

**Fig. 1:**
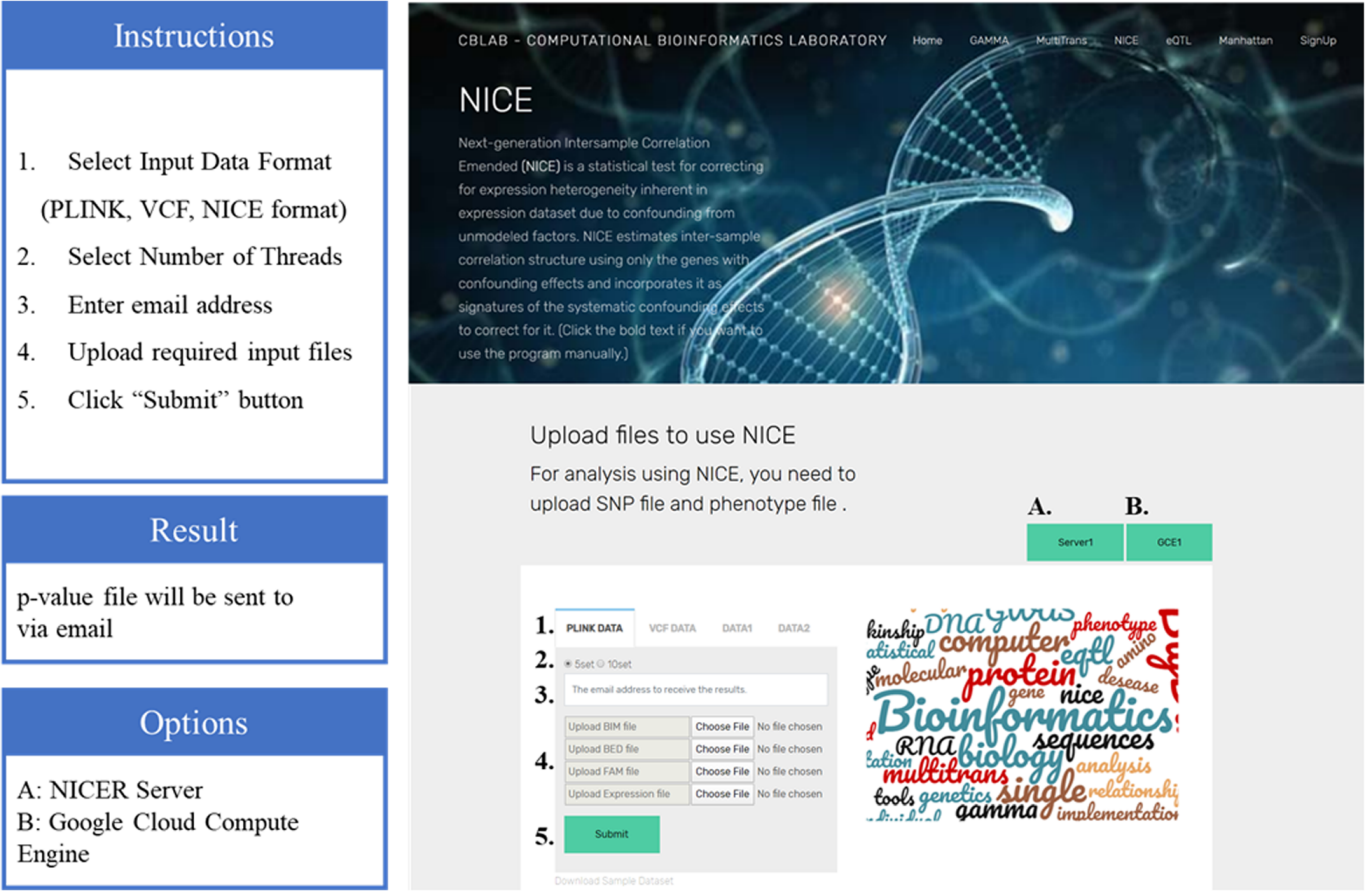
NICER website composition. Sequence of instructions to use NICER. (1) Select input data format among PLINK, VCF, and NICE format. (2) Select number of threads to use in the analysis. (3) Enter an email address. (4) Upload required input files. (5) Click submit button to start the analysis. Result file will be provided via email. Users can choose to run the analysis on either the NICER server (A) or GCE (B)

A majority of NICE users are biologists, who may not have the required experience in computer science to run the NICE program. NICER is created as a web-based tool, where users can activate an analysis by simply clicking on the web browser (Fig. 1). The execution is completely hidden from the user, and the user does not need to check each stage of running the program. After the analysis has been completed, NICER automatically sends a hyperlink to the email provided by the user. From the hyperlink, users can download the result file from the server. This means that after uploading the input data to NICER, users do not have to keep their computers running while performing the analysis and that they are notified of the results via e-mail.

### Google cloud compute engine

We allow users to run NICER on Google Compute Engine (GCE), which has several advantages as compared to an ordinary high-computing server. The GCE has more computational power to run programs with more threads. It is protected by Google, and suspicious activities are flagged and banned. As GCE is virtually created as a docker container, server hardware components such as CPU, RAM, and HDD can be flexibly modified, which is helpful to increase the number of parallel processes and shorten the execution time if needed. However, because the web service is uploaded to GCE, users may have to consider the sensitivity of the data and additional costs for using GCE, which they can avoid by using the free NICER on our server. For those who want to avoid the process burden of signing up to the Google Cloud service and installing NICER on it, we also provide a Google Cloud Compute Engine server link upon request.

### Downloadable NICER

For those who do not want to upload their data onto either our server or the GCE, we provide NICER as a downloadable version. The downloadable NICER is provided as a web software docker image file along with the source code. Users can easily download and install the docker image to run the NICER web service on their local computer or server with a few lines of code. Users do not need to worry about any initial preparation steps for running NICE such as building the running environment, installing specific packages required to run NICE program, creating a script file to run the NICE, or preparing required input data such as t-test statistics, as they are all incorporated and implemented inside the NICER program. NICER and its detailed documentation is provided on the NICER website http://cblab.dongguk.edu/NICER/NICE_index.jsp In addition, users are allowed to alter or update the source code to run alternative tests.

### Visualization tools

NICER includes graphic analysis tools to assist with interpreting analysis results. After the eQTL mapping, *p*-values are provided as a result. To identify the hotspots from the *p*-values, eQTL map is one of the most commonly used tools for the analysis. eQTL shows the strength of associations between every SNP (x-axis) and gene (y-axis) on 2-dimensional space that helps analyzing patterns of associations at a glance. A plot that shows the average of negative logarithm of *p*-values is another tool that can help with identifying regulatory hotspots from the *p*-values. NICER provides these plots as an additional service to the NICE program. Users can upload and use their own *p*-value tables as well. The results of the plot can be seen in a pop-up page, and the corresponding image is sent to the users’ personal e-mails for safekeeping.

### Input data format

NICER allows three input formats of genotype and phenotype for the analysis. In addition to the format for original NICE method, binary PLINK [11] format and VCF, which are the most commonly used formats in Genome-Wide Association Study (GWAS) and eQTL analysis are added. Since data format varies depending on the dataset, input format compatibility in NICER saves time to preprocess the data beforehand. Users can select their data format from the tab inside the website.

### Parallel processing

NICER runs on a high-performance computing server and enhances the analysis by implementing parallel processing. Within the NICE algorithm, the value of genome data is calculated in the units of SNPs and as the same algorithm is used for each unit, it could run separately for each SNP in parallel. NICER reduces the execution time by dividing the input matrix data into multiple sets and running them in parallel. As the NICE algorithm is complexly intertwined with different software for each stage, a multi-processing technique is used instead of multi-threading. Thus, the web server splits the input data into multiple sets and creates processes to run NICE, and the web engine keeps track of each process. Parallel processing shortens the execution time considerably as compared to the original NICE method.

## Results and discussion

### Performance analysis

NICER has shown significant improvement in terms of performance and user friendliness.

**Fig. 2:**
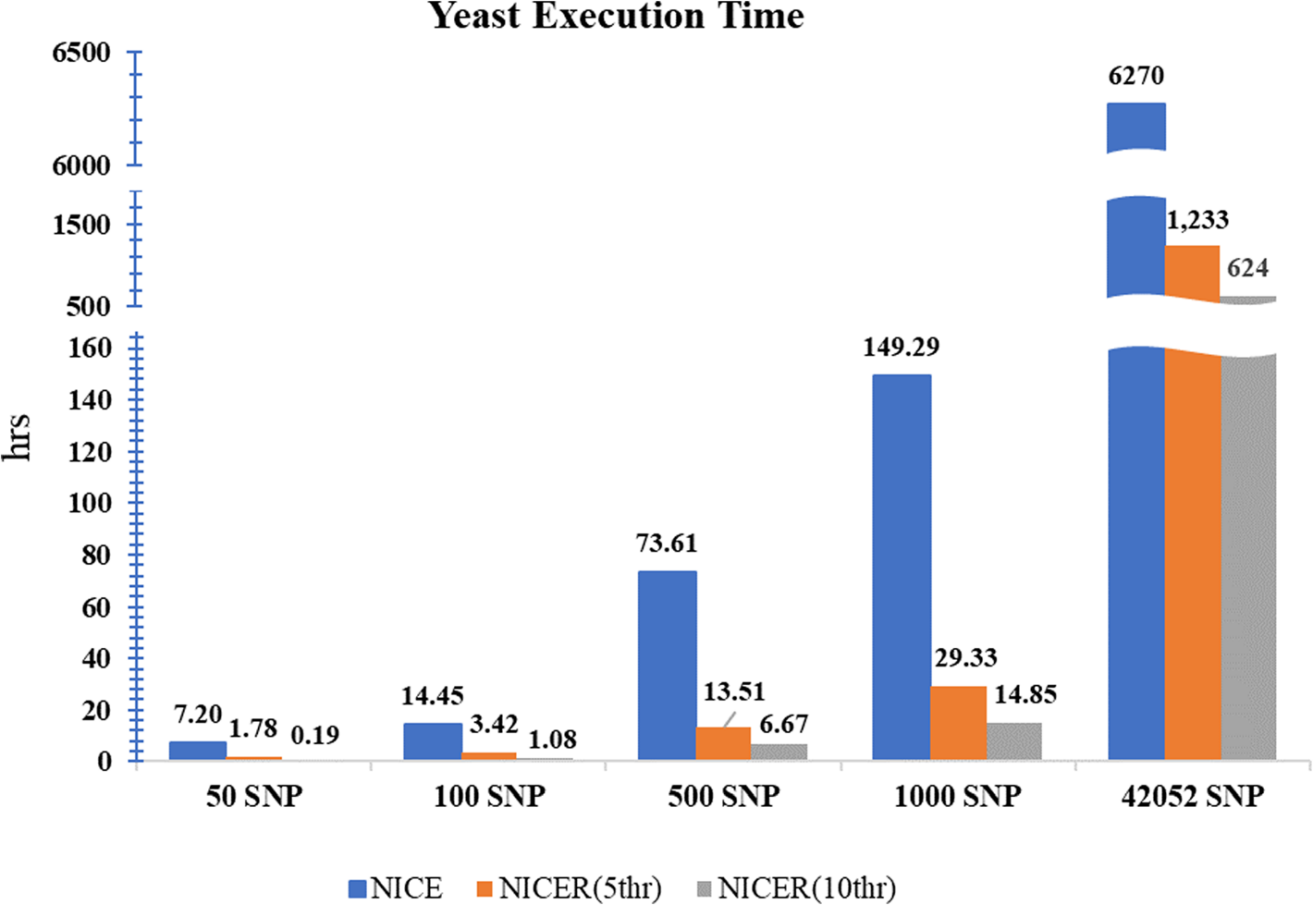
Time performance comparison between NICE and NICER. Using different numbers of threads, the x-axis corresponds to the number of SNPs analyzed and the y-axis corresponds the running time in hours. The yeast dataset is used for the test. The blue, orange, and gray bars show the performance of NICE, NICER using five processes, and NICER using 10 processes. The performance of the rightmost three bars are extrapolated from the results of 1000 SNPs

As shown in Fig. 2, the execution time decreases significantly when more threads are used. The results shown here are those obtained for an evaluation of a renowned yeast dataset [12]. The running time reduces linearly to the number of processes used; thus when the number of computing processes used is doubled, the execution time halves. Furthermore, the number of SNPs did not have any effect on the reduction of execution time, which could be attributed to multi-threading. NICER can reduce running time from 8 months to less than 3 days in an analysis of the yeast data of 42,052 SNPs and 5720 genes [12], utilizing 1000 processes in parallel.

### NICER analysis using the yeast dataset

**Fig. 3:**
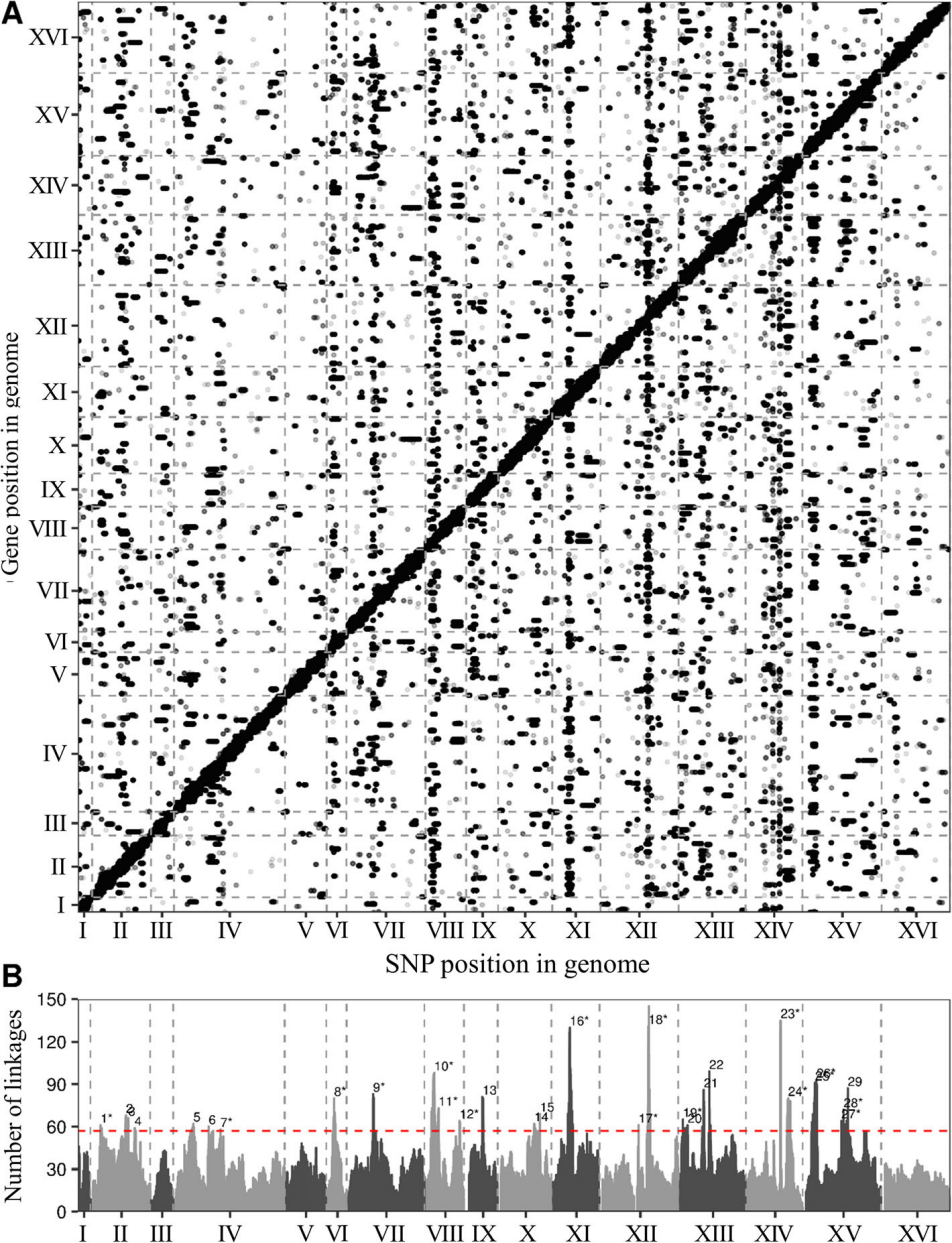
Hotspot identification of the yeast dataset. **a** The eQTL map showing the significant eQTLs across the whole genome. The x-axis corresponds to the SNP positions, and the y-axis corresponds to the gene positions. **b** The number of eQTLs (linkages) plotted in genome location. The numbers show the identified eQTL hotspots (Table 1). The dashed red line represents the threshold, and the asterisks indicate the previously identified hotspots [14–20]

We evaluated the proposed method by using a yeast dataset that contains 1012 meiotic segregants with 5720 genes and 42,052 SNPs [12]. After adjusting for batch effects and the growth covariate by using the ComBat method in the SVA R package [13], a significant number of eQTLs were identified on the basis of the NICER *p*-value of < 5 × 10^− 5^ (Fig. 3A). SNPs that regulate genes within 10 kb were defined as cis-eQTL.The thresholds for identifying the regulatory hotspots were based on a binominal test with the Bonferroni-corrected *p*-values of < 0.05/total number of SNPs (42,052). We estimated that the number of eQTL in each SNP followed a binomial distribution with the parameters *n* = total number of eQTLs except for *cis*-eQTLs across the whole genome and *p* = 1/42,052, which denotes the equal probability of eQTL in each SNP. If these SNPs had significantly more eQTLs than expected by chance, the genetic positions corresponding to the SNPs were defined as regulatory hotspots. To determine the putative regulators for a given hotspot, the *cis*-eQTL genes in the corresponding hotspot regions were regarded as the causal regulators. Next, we divided the whole genome of the Yeast into 602 20-kb bins to compare with the results of other studies related to Yeast eQTL hotspots [14–20]. If the neighboring bins had eQTL hotspots, then the bins were merged into a single bin and a gene. The results showed that among the 29 identified hotspots, 17 eQTL hotspots had been previously identified (Fig. 3 and Table 1).

**Table 1:** Results of hotspots identified by NICER using the yeast dataset. Putative regulators identified by previous studies are denoted in boldface [14–20]

eQTL hotspot	eQTL hotspot location	Putative regulator
1^*^	chrII:117298_A/G	**PIN4**, SAS3, MOH1, PTC3
2	chrII:466295_A/G	TKL2, ALG1, YBR109W-A, YBR116C
3	chrII:498623_C/T	CKS1, VMA2, MEO1, YBR126W-B, CCZ1
4	chrII:586961_C/T	UMP1, YPC1, EHT1, RPS6B, SMP1, FZO1
5	chrIV:266824_T/C	TMA17, QRI1, MSS2, QRI7, NSE4, TRM3
6	chrIV:476833_C/T	GCV1, DAS2
7^*^	chrIV:637663_A/T	YDR098C-B, **GIS1**, MSH6
8^*^	chrVI:96802_G/A	**FRS2**, LPD1, MDJ1, GNA1, **GAT1**, SMX2
9^*^	chrVII:362516_A/T	RPL7A, MPC1, HNM1, **AFT1**, SCY1, DBP3, YGL081W, YGL082W, MPS2
10^*^	chrVIII:114337_G/A	**GPA1**, **ERG11**, OSH7, QCR10, TDA3
11^*^	chrVIII:173637_G/T	DAP2, YHR033W, SLT2, **ERC1**, PUT2, PIH1, BRL1, RRM3
12^*^	chrVIII:463786_G/A	**STB5**, OYE2, YHR177W, YHR182W, GND1
13	chrIX:216335_C/T	SPO22, THS1, SER33, YIL077C, SEC28
14	chrX:489283_A/G	RAD26, CPR7, RAV1, RBH2
15	chrX:568363_G/A	MOG1, OPI3, HAM1, HOC1, NPA3, CDC11
16^*^	chrXI:232567_T/C	AAT1, HAP4, **KTI12**, YKL107W, APN1, SEG2, RAD27
17^*^	chrXII:514845_A/C	YLR179C, TFS1, VTA1, SAM1, TOS4, **YLR177W**, CBF5
18^*^	chrXII:650260_C/T	**HAP1**, MCP2
19^*^	chrXIII:46211_C/T	BUL2, **COQ5**, VAN1, YML108W, YML116W-A, DAT1, CTK3, TAF8, ZDS2
20	chrXIII:110807_T/C	CPR3, HMG1, YML079W, WAR1, YML082W, TDA9, DUS1
21	chrXIII:333449_G/A	RCH1, IMP2, MIH1, FAR8
22	chrXIII:409642_T/A	AVO2
23^*^	chrXIV:467219_A/G	**TOP2**
24^*^	chrXIV:571166_T/C	HHT2, SIW14, **CRZ1**
25^*^	chrXV:159827_A/G	**PHM7**
26^*^	chrXV:177015_A/G	**ATG19**, AVO1, ATP19, **IRA2**
27^*^	chrXV:520870_G/A	RAS1, PIN2, **RGS2**, **LEU9,** CRC1, KTR1, INP53, YOR105W, AM3, YOR111W
28^*^	chrXV:552069_C/T	**CAT5**, GCY1, LEO1, RTC5, PFY1
29	chrXV:610420_C/A	SPP2, RPB2, PNO1, ELG1, MDM32

## Conclusions

NICER is a user-friendly web application to effectively identify eQTL hotspots. By reducing the installation steps and hiding the complicated running steps from the users, NICER lowers the entry barrier from non-computer experts to use eQTL analysis. NICER can be easily accessed through the Internet and the NICER web service could be run not only on the top of the NICER server but also on the Google Compute Engine or users’ local computers with a very simple installing process. Moreover, NICER provides useful tools that visualize analysis results. Applying NICER on the yeast dataset, we effectively found many hotspots, where more than half of the hotspots contain putative regulators reported by previous studies elsewhere.

## Availability and requirements

Project name: NICER

Project home page: http://cblab.dongguk.edu/NICER/NICE_index.jsp

Operating system: CentOS7

Programming Language: Java, R

Other requirements: Apache Tomcat 9

License:

Any restrictions to use by non-academics: permission by the author

## Data Availability

Not applicable.

## Abbreviations

eQTL: Expression quantitative trait loci
GCE: Google Compute Engine
GWAS: Genome-Wide Association Study
NICE: Next-Generation Intersample Correlation Emended
SNP: Single-nucleotide polymorphisms
VCF: Variant call format
